# Tomatidine-stimulated maturation of human embryonic stem cell-derived cardiomyocytes for modeling mitochondrial dysfunction

**DOI:** 10.1038/s12276-022-00746-8

**Published:** 2022-04-04

**Authors:** Ye Seul Kim, Jung Won Yoon, Dasol Kim, Seunghak Choi, Hyoung Kyu Kim, Jae Boum Youm, Jin Han, Soon Chul Heo, Sung-Ae Hyun, Jung-Wook Seo, Deok-Ho Kim, Jae Ho Kim

**Affiliations:** 1 Department of Physiology, School of Medicine, Pusan National University, Yangsan, Republic of Korea; 2 Department of Physiology and Biophysics, College of Medicine, Cardiovascular and Metabolic Disease Center, Inje University, Busan, Republic of Korea; 3 Department of Oral Physiology, School of Dentistry, Pusan National University, Yangsan, Republic of Korea; 4 Safety Pharmacology Research Group, Korea Institute of Toxicology, Daejeon, 34114 Republic of Korea; 5 Department of Biomedical Engineering, Johns Hopkins University, Baltimore, MD 21205 USA

**Keywords:** Heart failure, Embryonic stem cells, Stem-cell differentiation

## Abstract

Human embryonic stem cell-derived cardiomyocytes (hESC-CMs) have been reported to exhibit immature embryonic or fetal cardiomyocyte-like phenotypes. To enhance the maturation of hESC-CMs, we identified a natural steroidal alkaloid, tomatidine, as a new substance that stimulates the maturation of hESC-CMs. Treatment of human embryonic stem cells with tomatidine during cardiomyocyte differentiation stimulated the expression of several cardiomyocyte-specific markers and increased the density of T-tubules. Furthermore, tomatidine treatment augmented the number and size of mitochondria and enhanced the formation of mitochondrial lamellar cristae. Tomatidine treatment stimulated mitochondrial functions, including mitochondrial membrane potential, oxidative phosphorylation, and ATP production, in hESC-CMs. Tomatidine-treated hESC-CMs were more sensitive to doxorubicin-induced cardiotoxicity than the control cells. In conclusion, the present study suggests that tomatidine promotes the differentiation of stem cells to adult cardiomyocytes by accelerating mitochondrial biogenesis and maturation and that tomatidine-treated mature hESC-CMs can be used for cardiotoxicity screening and cardiac disease modeling.

## Heart muscle: Promoting stem cell maturation yields a better model

A naturally occurring compound that promotes human heart muscle cell (cardiomyocyte) maturation could give researchers a more sophisticated model for studying drug toxicity. Researchers can use chemical signals to convert stem cells into functional cardiomyocytes, but the resulting cells lack key features of mature cells. Researchers led by Jae Ho Kim at Pusan National University, Yangsan, South Korea, have now demonstrated that treatment with the plant-derived compound tomatidine can coax such cells into a state that more closely reflects maturity. Tomatidine is known to prevent atrophy in skeletal muscle, and the researchers found that it also promotes appropriate levels of mitochondrial function and metabolic activity in human stem cell-derived cardiomyocytes. Tomatidine-treated cells also provided a superior system for modeling drug-induced heart damage, and could thus help signal potential cardiotoxicity during drug development.

## Introduction

Cardiotoxicity is one of the leading causes of drug withdrawal, and cardiotoxicity testing is essential for early toxicity screening during drug development^1^. Traditionally, cardiotoxicity analysis is conducted either in noncardiac cells overexpressing specific ion channels or in in vivo animal models. However, the use of these screening methods is hampered by their inability to predict cardiotoxicity, which is primarily caused by species differences and the lack of cardiomyocyte (CM)-specific signaling components in these systems^2^. The lack of a human CM cell line or difficulty in preparing primary human CMs has also been a major impediment to drug development and analysis of cardiotoxicity.

Human pluripotent stem cells, such as embryonic stem cells and induced pluripotent stem cells, have been utilized to produce functional CMs, which are a good model for disease modeling, drug screening, and cardiotoxicity testing^3^. Temporal application of a glycogen synthase kinase 3 inhibitor combined with a Wnt inhibitor was shown to be sufficient to produce functional CMs^4^. Although human pluripotent stem cell-derived CMs (hPSC-CMs) have structural and functional properties resembling those of adult CMs, they have been reported to exhibit immature phenotypes compared to adult CMs^5^. hPSC-CMs exhibit a less organized sarcomeric structure; have a lower maximum contractile force, slower upstroke velocity, and higher resting membrane potential; do not contain T-tubules; and have reduced mitochondrial content and function^6,7^. These factors highlight the necessity of using mature hPSC-CMs for the modeling of cardiovascular diseases and cardiotoxicity analysis. An increasing body of evidence suggests that several chemotherapeutic drugs, including doxorubicin (Dox), trastuzumab, and sunitinib, exhibit cardiotoxicity mainly associated with mitochondrial damage^8^. For instance, Dox specifically binds to cardiolipin, which plays a key role in mitochondrial crista organization through the formation of cardiolipin-based lipid-protein microdomains^9^, leading to selective mitochondrial accumulation of Dox^10,11^ and induction of cell death by increased mitochondrial reactive oxygen species (ROS) production^12^. Moreover, relatively mature CMs could better model Dox-induced cardiotoxicity than immature CMs^13^, supporting the efficacy of mature CMs in cardiotoxicity research.

Tomatidine is a metabolite of α-tomatine, which is abundant in unripe green tomatoes and exhibits various activities, including antibiotic, anti-inflammatory, and anticarcinogenic effects^14,15^, in cells. Tomatidine has been identified as a natural compound that inhibits skeletal muscle atrophy *via* a system-based discovery strategy^16^. In cultured skeletal myotubes, tomatidine treatment stimulated mTORC1 signaling and anabolism, leading to the accumulation of protein and mitochondria and cell growth. Furthermore, tomatidine has been found to reduce age-related skeletal muscle weakness and atrophy by inhibiting activating transcription factor 4 (ATF4)^17^, a bZIP transcription factor subunit that regulates oxidative and other stress responses^18^. In addition, tomatidine treatment was reported to induce mitophagy of mitochondria and have beneficial effects through the SKN-1/Nrf2 signaling system in *Caenorhabditis elegans*^19^. However, the effects of tomatidine on CM differentiation, maturation from PSCs, and mitochondrial functionality have not been investigated.

In this study, we examined the effects of tomatidine on CM differentiation and maturation from human embryonic stem cells (hESCs). In addition, we investigated the effects of tomatidine treatment on mitochondrial phenotypes and function in hESC-derived cardiomyocytes (hESC-CMs). The present study demonstrates, for the first time, that tomatidine treatment induces the differentiation of hESC-CMs to more mature CMs with increased mitochondrial mass and function, which serve as a highly useful platform for the investigation of cardiotoxicity.

## Materials and methods

RPMI1640 (#LM011-01) and D-PBS (#LM-001-01) were purchased from Welgene (Gyeongsan-si, Gyeongsanbuk-do, Republic of Korea). mTeSR1 (#85850) was purchased from STEMCELL Technologies (Vancouver, Canada). DMEM/F12 (#11330-032), B27 minus insulin (#A18956-01), B27 (#17504-044), nuclease-free water (#AM9938), glucose-free DMEM (#11966-025), 0.05% trypsin-EDTA (#25300–054), 0.5 M EDTA (#15575-020), Accutase (#A11105-01), dispase (#17105-041), fetal bovine serum (#16000-044), MitoSOX Red mitochondrial superoxide indicator (#M36008), potential-sensitive ANEP dyes (Di-8-ANEPPS; #D3167), tetramethylrhodamine (TMRM; #T668), and SERCA2 ATPase (#MA3919) were purchased from Thermo Fisher Scientific (Waltham, MA, USA). Matrigel (#354277) was purchased from Corning (New York, NY, USA). Y27632 (#Y0503), Hank’s balanced salt solution (#4891), tomatidine (#T2909), α-sarcomeric actinin (α-SA; #A7732), gelatin (#G9391), BSA (#A7946), and dimethyl sulfoxide (DMSO; #D5879) were purchased from Sigma-Aldrich (St. Louis, MO, USA). L-lactic acid (#129-02666) was purchased from Fujifilm Wako Pure Chemical Corporation (Osaka, Japan). GAPDH (#SC-47724) was purchased from Santa Cruz Biotechnology (Dallas, TX, USA). Cardiac troponin T (cTnT; #ab45932), cardiac troponin I (cTnI; #ab47003), BIN1 (#ab185950), OPA1 (#ab42364), and mic60 (#ab110329) were purchased from Abcam PLC (Cambridge, UK). MitoTracker Green FM (#9074) was purchased from Cell Signaling Technology, Inc. (Danvers, MA, USA). Myosin light chain 2a (MLC2a; #311 011) was purchased from Synaptic Systems (Goettingen, Germany). Myosin light chain 2v (MLC2v; #10906-1-AP) was purchased from Proteintech Group, Inc. (Rosemont, IL, USA). Junctophilin-2 (JPH2; #orb13188) was purchased from Biorbyt, Ltd. (Cambridge, UK). CHIR99021 (#CT-99021) was purchased from Selleck Chemicals (Houston, TX, USA). IWP2 (#3533) was purchased from Tocris Bioscience (Bristol, UK). SR 28292 was purchased from Cayman Chemical (#22084). The PGC1-alpha (#NBP1-04676) antibody was purchased from Novus Biological (Centennial, CO, USA). Tom20 (#sc17764) was purchased from Santa Cruz Biotechnology, Inc. (Dallas, TX, USA). Ursolic acid was purchased from Tokyo Chemical Industry Co., Ltd. (TCI, Tokyo, Japan). Information about the antibodies used in this study is listed in Supplementary Table 1.

### Culture of hESCs and differentiation to cardiomyocytes

The hESC line H9 was purchased from the WiCell Research Institute (Madison, Wisconsin, USA) and maintained as described previously. hESCs were maintained on Matrigel (Corning, #354277) in Essential 8 medium (Thermo Fisher Scientific) and passaged every 4 days using 0.5 mM EDTA. Cardiomyocyte differentiation was performed as previously reported with slight modifications^4^. The detailed experimental procedures for cardiomyocyte differentiation and structural and functional characterization of the hESC-CMs and all the other materials and methods used in this study are described in the Expanded Materials and Methods in the online supplement file.

### Culture of hESCs and differentiation to cardiomyocytes

The hESC line H9 was purchased from the WiCell Research Institute (Madison, Wisconsin, USA) and maintained as described previously. hESCs were maintained on Matrigel (Corning, #354277) in Essential 8 medium (Thermo Fisher Scientific) and passaged every 4 days using 0.5 mM EDTA. Cardiomyocyte differentiation was performed as previously reported with slight modifications^4^. In brief, H9 hESCs were seeded on Matrigel in mTeSR1 medium and differentiated to CMs by treatment with 12 μM CHIR99021 for 24 h in RPMI medium with B27 minus insulin. On Day 3, the cells were treated with 5 μM IWP2 for 48 h and replaced in medium without chemicals every 48 h until Day 8. Tomatidine treatment was started on Day 5. The medium was replaced with RPMI with B27 at Day 8 of differentiation and every 48 h. For increased cardiomyocyte purity, hESC-CMs were selected for subsequent glucose starvation two times (two days per time) at Day 20. Following this protocol, hESC-CMs were reseeded for 24 h with 10 μM Y27632 in 0.1% gelatin-coated plates. The culture media was refreshed every 2 days and maintained at 37 °C and 5% CO_2_.

### Measurement of cellular respiration

Mitochondrial respiration was measured using an Agilent Seahorse XFp analyzer (Agilent Seahorse, Santa Clara, CA, USA) and Seahorse XF Cell Mito Stress Test Kit (#103010-100). The test was performed following the manufacturer’s protocol. hESC-CMs were seeded on a 0.1% gelatin-coated XFp cell culture plate at 15,000 cells/well in RPMI containing 20% FBS and maintained for 3 days in RPMI/B27 medium. The day before the experiment, a sensor cartridge was hydrated in an XF calibrant at 37 °C in a non-CO_2_ incubator. The XF assay medium was supplemented with 1 mM sodium pyruvate, 4 mM L-glutamine, and 10 mM D-(+)-galactose at pH 7.4 and 37 °C. One hour before the analysis, the hESC-CMs were replenished with XF assay medium after washing with XF medium. The cells were then incubated in a non-CO_2_ incubator at 37 °C. Thirty minutes before the analysis, 2 μM oligomycin, 0.5 μM FCCP, and 2 μM each of a mix of antimycin A/rotenone was loaded into the A, B, and C injection ports of a hydrated sensor cartridge with 20 μL, 22 μL, and 25 μL of a 10× solution of components in the XF Mito Stress Test kit. After the calibration was completed, the cell culture plate was injected into the instrument.

### Statistical analysis

All grouped data are presented as the mean ± S.D. (*n* ≥ 3, independent groups) determined by Bonferroni post-tests for comparison. Statistical significance was determined using one-way ANOVA, two-way ANOVA, and two-tailed unpaired Student’s *t*-tests. All statistical analyses were performed with GraphPad Prism version 5.03.

## Results

### Tomatidine treatment stimulates structural maturation of hESC-CMs

**Fig. 1:**
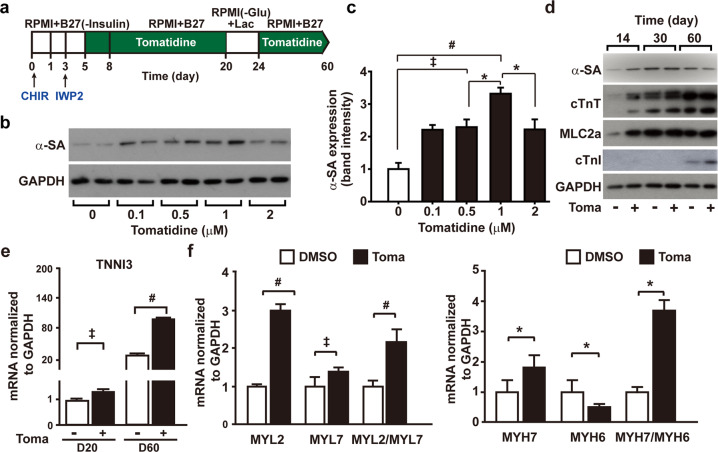
Stimulatory effects of tomatidine treatment on CM differentiation of human embryonic stem cells. **a** Schematic protocol of tomatidine treatment during CM differentiation of H9 cells. The period for tomatidine treatment is indicated in green. On Day 20, hESC-CMs were selected by lactate metabolic selection for 96 h. **b** Dose-dependent effects of tomatidine on the expression of α-SA. Western blot images of α-SA and GAPDH are shown. **c** Quantification of the expression of α-SA in panel (**a**). The band intensities of α-SA were normalized to those of GAPDH. **d** Time-dependent effects of tomatidine treatment on CM differentiation of hESCs. During CM differentiation of H9 cells in the absence or presence of 1 μM tomatidine, the protein levels of α-SA, cTnT, MLC2a, cTnI, and GAPDH were probed with western blotting at the indicated times. **e** Effects of tomatidine treatment on the mRNA expression of *TNNI3* on Days 20 and 60 in the control CMs and Toma-CMs. **f** Effects of tomatidine treatment on the mRNA expression of *MYL2, MLY7, MYH6*, and *MYH7*. hESC-CMs were treated with vehicle or 1 μM tomatidine, and the relative mRNA levels of the genes were determined on Day 60. Data are shown as the mean ± S.D. (*n* = 5). *p < 0.05; ^‡^*p* < 0.01; ^#^*p* < 0.001.

To induce CM differentiation of hESCs, we adopted a previously reported differentiation protocol based on sequential activation of the Wnt pathway by GSK3 inhibition using CHIR99021, followed by Wnt inhibition using IWP2^4^. To examine the effects of tomatidine treatment on CM differentiation, we treated hESCs with tomatidine after IWP2 treatment, except during the lactate metabolic selection period (Fig. 1a). We treated the cells with increasing concentrations of tomatidine and analyzed the expression of α-sarcomeric actinin (α-SA), a cardiomyocyte-specific protein, using western blotting on Day 20. Treatment with 0.5 μM tomatidine significantly increased α-SA expression, and the maximal increase was obtained at 1 μM (Fig. 1b and c). Therefore, 1 μM tomatidine was used in the subsequent experiments. Prolonged incubation of hESC-CMs with tomatidine accelerated spontaneous increases in cardiac structural proteins, including cardiac troponin T (cTnT) and myosin light chain 2 isoforms (MLC2a) (Fig. 1d). In particular, the expression of cTnI, which is a key signature of the maturation isoform, significantly increased in the 60-day culture with tomatidine treatment compared to that of the control hESC-CMs (control CM) (Fig. 1d). Flow cytometry showed similar expression profiles of CM markers in the lactate-selected culture of CMs after tomatidine treatment but increased expression after 2 weeks under tomatidine treatment (Supplementary Fig. 1). Next, we determined the mRNA levels of other CM markers, including cTnI (*TNNI3*), myosin light chain 2 (*MYL2* or *MLC2v*), myosin light chain 7 (*MYL7* or *MLC2a*), the atrial isoform myosin heavy chain 6 (*MYH6* or *α-MHC*), and the ventricle isoform myosin heavy chain 7 (*MYH7* or *β-MHC*), upon tomatidine treatment. TNNI3, MYL2, and MYH7 expression increased with tomatidine treatment (Fig. 1e, f, and Supplementary Table 2). Moreover, tomatidine treatment increased the ratios of *MYL2/MYL7* and *MYH7/MYH6* in the hESC-CMs (Fig. 1f). These results suggest that tomatidine treatment enhances the differentiation efficiency and structural maturation of hESC-CMs.

### Tomatidine treatment enhances cardiac hypertrophy and organization of sarcomeric structure

**Fig. 2:**
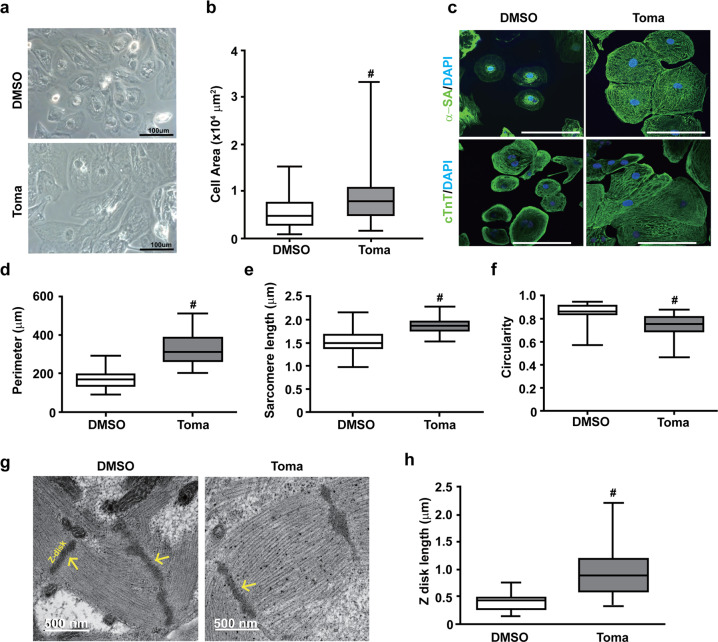
Effects of tomatidine treatment on the morphology of human embryonic stem cell-derived cardiomyocytes. **a**, **b** Effects of tomatidine treatment on the morphology of hESC-CMs. **a** Bright field images of the control CMs and Toma-CMs on Day 30. Scale bar = 100 μm. **b** Quantification of the cell area of the control CMs and Toma-CMs. **c**–**f** Immunocytochemistry images (**c**) and quantification of the measurements of the perimeter (**d**), sarcomere length (**e**), and circularity (**f**) of the control CMs and Toma-CMs (*n* = 77). Representative TEM images (**g**) and quantification of the measurement of the z disk length (**h**) in the control CMs and Toma-CMs. Data are shown as the mean ± S.D. (*n* = 33). *p < 0.05; ^‡^*p* < 0.01; ^#^*p* < 0.001.

Morphological changes that occur during the maturation of CMs from a fetal to an adult phenotype include a marked increase in cell size and changes in cell shape from round to rod-like^20^. To explore the effects of tomatidine treatment on morphological changes, we analyzed the 60-day culture of hESC-CMs in further experiments. Analysis of bright field images showed that tomatidine treatment prominently increased individual CM size (Fig. 2a and b). Compared to the control CMs, the Toma-CMs showed a highly organized sarcomeric structure throughout the cell, as verified by the expression of α-SA and cTnT (Fig. 2c). Moreover, the Toma-CMs exhibited an increase in cell perimeter and sarcomere length and a decrease in the circularity index, recapitulating the characteristics of mature CMs (Fig. 2d–f). The sarcomere lengths of the control CMs and the Toma-CMs were approximately 1.5 ± 0.3 and 1.9 ± 0.2 μm, respectively. Electron microscopy revealed that the Toma-CMs exhibited mature ultrastructural features with better-aligned myofibrils capable of strong force generation, and the length of the Z-disk was significantly increased by tomatidine treatment (Fig. 2g and h). These results indicate that tomatidine stimulates physiological hypertrophy and sarcomere maturation.

### Tomatidine promotes T-tubule formation in hESC-CMs

**Fig. 3:**
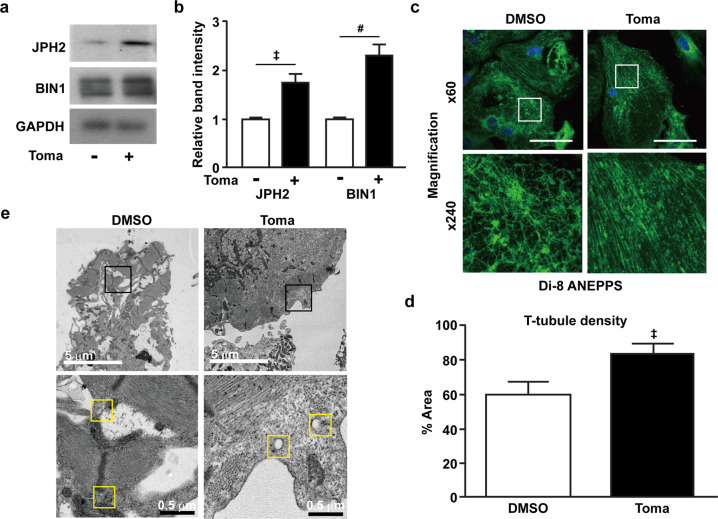
Effects of tomatidine treatment on T-tubule formation in human embryonic stem cell-derived cardiomyocytes. **a**, **b** Immunoblotting images (**a**) and quantification of JPH2 and BIN1 expression (**b**) in hESC-CM. **c** Immunofluorescence staining of T-tubules with Di-8-ANEPPS dye in the control CMs and Toma-CMs. **d** Quantification of the T-tubule density in panel (**c**). **e** TEM images showing T-tubule-like structures in the control CMs and Toma-CMs. Data are shown as the mean ± S.D. (*n* = 3–10). *p < 0.05; ^‡^*p* < 0.01; ^#^*p* < 0.001.

Transverse tubules (T-tubules) are part of the CM membrane that carries electrical impulses. During CM maturation, T-tubule development is essential for the establishment of excitation-contraction coupling^21^. To examine whether tomatidine treatment could improve the development of T-tubules, we analyzed the expression of bridging integrator 1 (BIN1)^22,23^ and junctophilin-2 (JPH2), the key proteins in T-tubule biogenesis and subsequent dyad formation^24^, in hESC-CMs on Day 60. Western blot analyses confirmed that the expression of JPH2 and BIN1 in the Toma-CMs was greater than that in the control CMs (Fig. 3a and b). The staining of hESC-CMs with Di-8-ANEPPS, which is a voltage-sensitive plasma membrane lipid dye, revealed that the Toma-CMs showed a highly organized and periodic pattern of Di-8-ANEPPS staining with regular spacing compared with the control CMs (Fig. 3c), further indicating increased T-tubule density (Fig. 3d). Figure 3e shows transmission electron microscopy (TEM) images of T-tubules in hESC-CMs, with the Toma-CMs exhibiting a more developed T-tubule structure than the control CMs. Overall, these results suggest that tomatidine treatment promotes T-tubule development in hESC-CMs.

### Tomatidine treatment increases mitochondrial biogenesis and metabolism in hESC-CMs

**Fig. 4:**
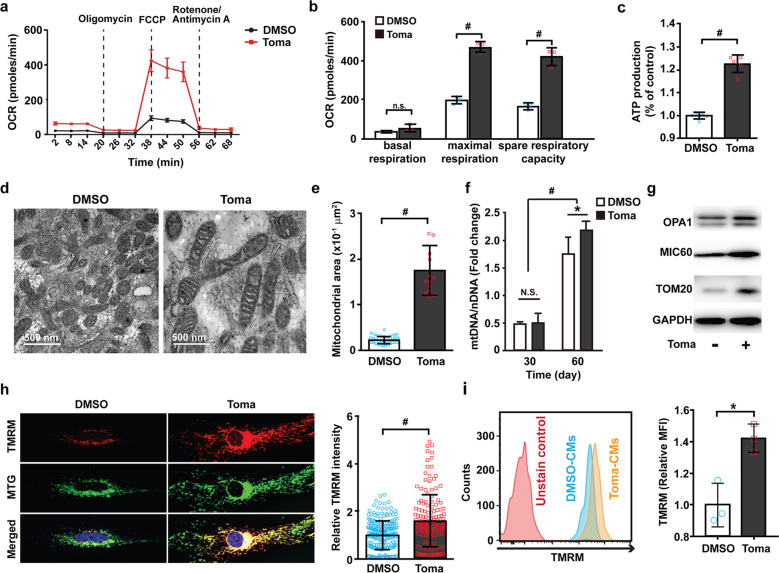
Tomatidine treatment stimulates metabolic maturation in human embryonic stem cell-derived cardiomyocytes. **a** Representative data of mitochondrial oxygen consumption rate analysis using the Agilent Seahorse XFp analyzer in the control CMs and Toma-CMs (*n* = 3). **b** Basal respiration, maximal respiration, and spare respiratory capacity were quantified from panel (**a**). **c** Measurement of ATP levels in the control CMs and Toma-CMs using a CellTiter-Glo kit (*n* = 30). **d** TEM images of mitochondrial structure in the control CMs and Toma-CMs. Scale bar = 500 nm. **e** Quantification of mitochondrial area in the control CMs and Toma-CMs. The mitochondrial area was quantified from panel (**d**) (*n* = 14–33). **f** The effects of tomatidine treatment on the ratio of mtDNA per nDNA in hESC-CMs. The amounts of mtDNA and nDNA were measured with qRT-PCR at Days 30 and 60 in the control CMs and Toma-CMs, and the ratio of mtDNA per nDNA was calculated. (*n* = 3). **g** Immunoblotting of cell lysates with antibodies against mitochondrial (OPA1, MIC60, TOM20) and cytosolic (GAPDH) proteins in the control CMs and Toma-CMs. **h** Immunofluorescence analysis of mitochondrial membrane potential activity with TMRM dye (*n* = 120). **i** Representative flow cytometry plot (left panel) and quantification of TMRM intensity (*n* = 3) (mean fluorescence intensity, MFI, right panel). Data are shown as the mean ± S.D. *p < 0.05; ^‡^*p* < 0.01; ^#^*p* < 0.001.

Recent studies have shown that mitochondrial metabolism plays a key role in CM maturation^25^. In particular, maximal mitochondrial oxygen uptake has been used as an indicator of cardiomyocyte mitochondrial maturation^26^. To test this hypothesis, we performed a Mito Stress assay to determine the effect of tomatidine treatment on the energy metabolism of hESC-CMs (Fig. 4a). In the Toma-CMs, there was no significant change in basal respiratory volume compared with that in the control CMs. However, the maximum respiration capacity and spare respiratory capacity, which are the maximum values of oxygen that can be ingested during contraction and relaxation, showed a significant increase (Fig. 4b). Furthermore, ATP production was substantially enhanced in the Toma-CMs compared with the control CMs (Fig. 4c). To determine whether the number and structure of mitochondria are affected by tomatidine treatment, we analyzed the mitochondria in hESC-CMs using a transmission electron microscope. In the Toma-CMs, mitochondria had a clear crista edge with a thicker mitochondrial membrane. Overall, the length and size of the mitochondria were longer and larger in the Toma-CMs than in the control CMs (Fig. 4d and e). The ratio of mitochondrial DNA per genomic DNA in the Toma-CMs was significantly increased compared to that in the control CMs (Fig. 4f, Supplementary Table 3). Furthermore, the levels of mitochondrial proteins, including OPA1, MIC60, and Tom20, were increased in the Toma-CMs compared with the control CMs (Fig. 4g). Interestingly, the expression of MIC60, a mitochondrial inner membrane complex protein, drastically increased in response to tomatidine treatment compared with that of TOM20, a subunit of the TOM complex located in the outer mitochondrial membrane. This result is consistent with the tomatidine-induced development of crista structure in mitochondria. The mitochondrial membrane potential acts as a force to form ATP in the mitochondrial ATP synthase complex. We performed TMRM staining to determine whether tomatidine treatment also affects the mitochondrial membrane potential in hESC-CMs. Compared with that in the control CMs, the intensity of TMRM dye was significantly enhanced in the Toma-CMs, as demonstrated by immunostaining and flow cytometry (Fig. 4h and i), suggesting a tomatidine-induced increase in mitochondrial membrane potential in hESC-CMs. Moreover, the generation of mitochondrial ROS was significantly enhanced in the Toma-CMs compared with the control CMs (Supplementary Fig. 2). These results suggest that tomatidine treatment enhances the content and function of mitochondria in hESC-CMs.

### Tomatidine induces maturation of electrophysiological properties in hESC-CMs

**Fig. 5:**
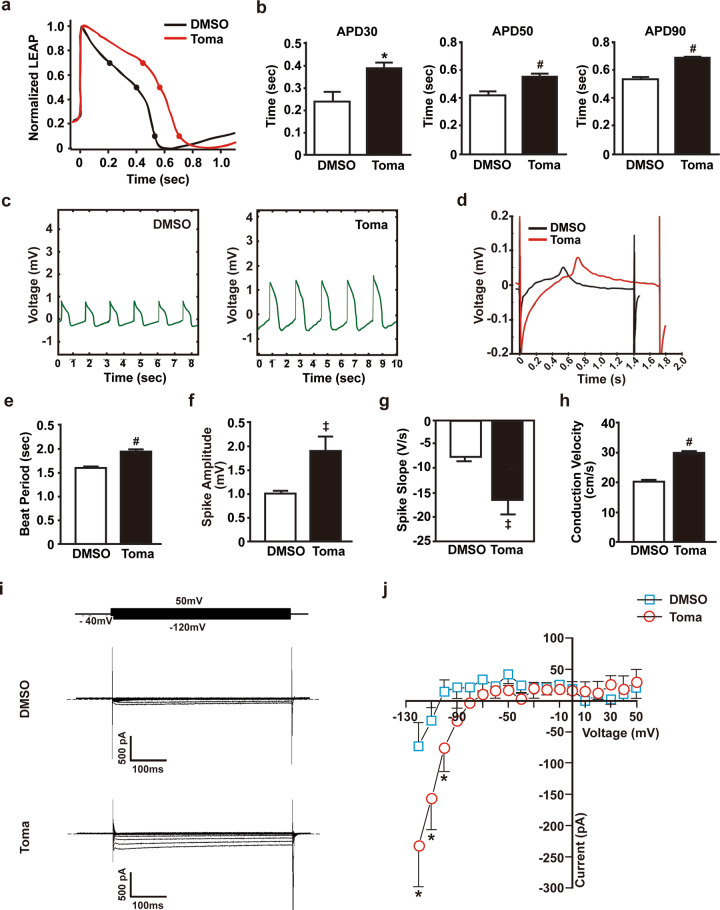
Electrophysiological properties of tomatidine-treated cardiomyocytes. **a** Representative trace of the change in action potential duration during axon cardiac LEAP analysis. **b** Quantification of APD30, 50, and 90 in the control CMs and Toma-CMs. **c** Representative beat rate trace of the control CMs and Toma-CMs. **d** Representative trace of the change in field potential duration during axon cardiac field potential analysis. Quantification of the change in beat period (**e**), spike amplitude (**f**), spike slope (**g**), and conduction velocity (**h**) in the Day 60 control CMs and Toma-CMs. Data are shown as the mean ± S.D. (*n* = 3). **i** Inward-rectifying K^+^ currents in the control CMs and Toma-CMs. Whole-cell membrane currents were obtained by applying 500 ms test pulses between −120 and 50 mV from a holding potential of −40 mV (see the inset). The Ba^2+^-sensitive component of whole-cell membrane currents. **j** Current-voltage relationship of Ba^2+^-sensitive currents in the control CMs and Toma-CMs. Representative membrane currents (**i**) and current-voltage relationships (**j**) were all obtained by averaging the results from seven different cells. Data are shown as the mean ± S.D. *p < 0.05; ^‡^*p* < 0.01; ^#^*p* < 0.001.

To confirm the stimulatory effects of tomatidine treatment on the maturation of hESC-CMs, we measured the electrophysiological properties using a microelectrode array. The Toma-CMs exhibited a local extracellular action potential (LEAP) signal resembling the mature cardiac action potential compared with the control CMs (Fig. 5a). The Toma-CMs showed a longer action potential duration (APD30, APD50, and APD90) than the control CMs (Fig. 5b). Compared with the control CMs, the Toma-CMs showed a reduction in spontaneous beating frequency and an increase in spike potential (Fig. 5c). Consistently, the field potential traces of the Toma-CMs exhibited longer spontaneous beating periods, increased spike amplitude and conduction velocity, and reduced spike slope mean (Fig. 5d–h). Calcium handling plays a key role in the regulation of contraction and relaxation in CMs^27^. The Toma-CMs exhibited increases in peak amplitude, Vmax decay, and time to decay of Ca^2+^ spikes (Supplementary Fig. 3). The mRNA levels of cardiac ion channels implicated in the generation of action potential and calcium influx in mature CMs, such as *SCN5A*, *CACNA1C*, *KCNJ2*, and *KCNJ12*, were increased in the hESC-CMs treated with tomatidine (Supplementary Fig. 4). The inward rectifying K^+^ channels Kir2.1 (*KCNJ2*) and Kir2.2 (*KCNJ12*) primarily maintain the resting membrane potential in adults^28^. To explore whether tomatidine treatment affects the inward rectifying K^+^ currents in hESC-CMs, we measured whole-cell membrane currents and Ba^2+^-sensitive currents using patch-clamp analysis and observed higher Ba^2+^-sensitive inward rectifying K^+^-currents in the Toma-CMs than in the control CMs (Fig. 5i, Supplementary Fig. 5). The voltage-current relation exhibited significantly greater inward-rectifying K^+^-currents, I_k1_, in the Toma-CMs than in the control CMs (Fig. 5j). These results suggest that tomatidine induces the maturation of electrophysiological characteristics of hESC-CMs in adult CMs.

### The Toma-CMs exhibit increased sensitivity to Dox-induced cardiotoxicity

**Fig. 6:**
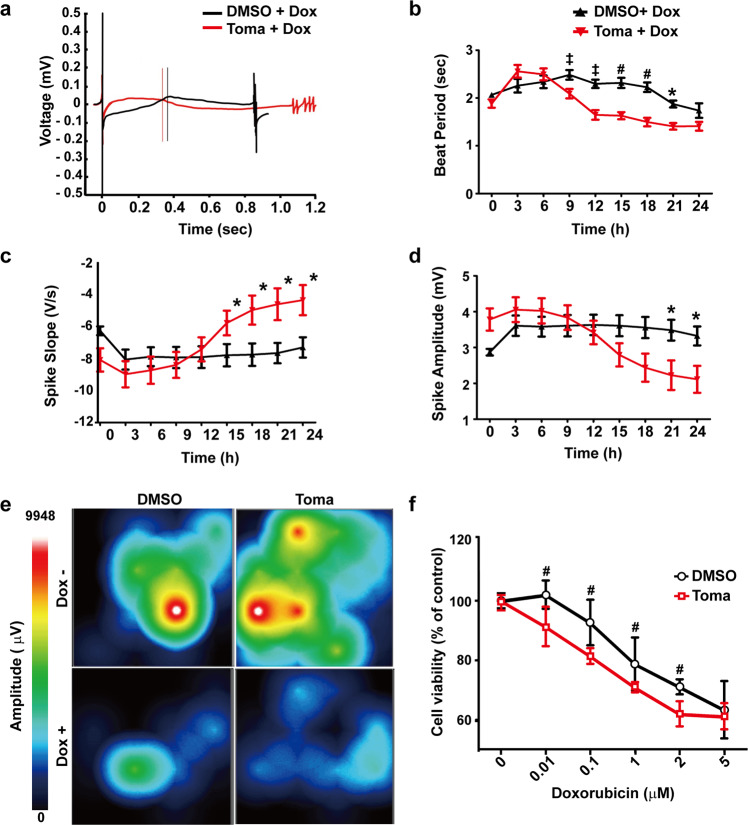
Doxorubicin-induced cardiotoxicity in tomatidine-treated cardiomyocytes. **a** Representative multielectrode array traces of cardiac electrical activities in the control CM and Toma-CM monolayers. Representative beat interval (**b**), spike slope (**c**), and spike amplitude (**d**) traces of the control CMs and Toma-CMs (*n* = 6, independent biological replicates are presented). **e** Activation map and example electro signals from the control CMs and Toma-CMs showing the direction of the field potential spreading throughout the cell monolayer with/without doxorubicin. **f** Cell viability assays detected a decrease in ATP levels following induction of mitochondrial dysfunction with doxorubicin for 12 h (*n* = 3–10). Data are shown as the mean ± S.D. *p < 0.05; ^‡^*p* < 0.01; ^#^*p* < 0.001.

Dox is a well-known anticancer drug^29,30^. However, it has been reported to induce acute cardiotoxicity in up to 11% of patients^31–34^. To evaluate the efficacy of the Toma-CMs in cardiotoxicity testing, we measured the effects of Dox treatment on the electrophysiological properties of the Toma-CMs and control CMs using multielectrode array analysis (Fig. 6a). The Toma-CMs began to exhibit a reduction in the beat period, which corresponds to the arrhythmia phenomenon, 12 h after Dox treatment. Dox-induced arrhythmia occurred earlier in the Toma-CMs than in the control CMs (Fig. 6b). Furthermore, the Toma-CMs exhibited an increased spike slope and reduced spike amplitude in response to Dox treatment compared with the control CMs (Fig. 6c and d). An electromap for the optical mapping of hESCs recorded the spread of activation during the sinus rhythm (Fig. 6e). The map marked phase discontinuities in response to treatment with Dox in both groups. We found that the Toma-CMs exhibited increased sensitivity to Dox-induced cell death compared with the control CMs (Fig. 6f), suggesting the efficacy of the Toma-CMs for Dox-induced cardiotoxicity analysis.

We next measured the effects of Dox treatment on mitochondrial membrane potentials and ROS, which were probed by TMRM and MitoSOX staining, respectively. Although Dox treatment slightly increased the mitochondrial membrane potential and ROS production in the control CMs, the Toma-CMs exhibited substantially increased mitochondrial membrane potentials and ROS levels (Supplementary Fig. 6). Taken together, these results suggest that increased mitochondrial membrane potential and ROS may be implicated in the sensitization of the Toma-CMs to Dox-induced cardiotoxicity.

## Discussion

In the present study, we demonstrated that tomatidine stimulated the differentiation of hESCs to mature CM-like phenotypes. Previously, tomatidine was shown to enhance lifespan through mitophagy via the SKN-1/Nrf2 pathway in *C. elegans*^19^. Moreover, tomatidine has been reported to suppress skeletal muscle atrophy by activating the mTORC1 pathway and reducing ATF4 activity^16,17^. mTORC1 activates the translation of proteins used to build muscle and plays an essential role in the hypertrophic growth of CMs after the early postnatal period^35^. In addition, ursolic acid, a natural triterpene, has been shown to reduce muscle atrophy^36^ and increase mitochondrial mass and ATP generation through activation of the AMPK and PGC-1 pathways in skeletal muscle^37^. We found that both tomatidine and ursolic acid stimulated α-SA expression, mitochondrial membrane potential, and ROS (Supplementary Fig. 7). Moreover, treatment with the PGC1α blocker SR18292 abrogated the tomatidine-induced expression of the CM-specific markers cTnT and MLC2v in hESC-CMs (Supplementary Fig. 8). These results suggest that the natural compounds tomatidine and ursolic acid accelerate CM maturation by stimulating muscle hypertrophy and mitochondrial biogenesis *via* a PGC1α-dependent mechanism.

Tomatidine treatment increased the expression levels of CM-specific contractile proteins such as cTnT and MLC2a. Furthermore, the expression levels of mature CM markers, including cTnI, MYL7, MYH6, and T-tubule components, were augmented by tomatidine treatment. The Toma-CMs exhibited increased cell size, sarcomere length, and T-tubule density. The maturation of differentiated CMs was shown to be stimulated by long-term culturing and biophysical stimuli, including substrate stiffness, cell patterning, and electrical stimulation^20^. Moreover, various extracellular stimuli, such as triiodothyronine (T3), dexamethasone, insulin-like growth factor-1, and microRNAs, including let-7, have been reported to enhance the maturation of differentiated CM^38–41^. In addition, coculture of CMs with noncardiomyocytes was shown to promote the maturation of CMs in vitro^42,43^, and culture of differentiated CMs in the presence of fatty acids as an energy source enhanced the maturation of CMs^44^. We found that tomatidine treatment increased the expression of cTnI and cTnT as potently as fatty acid treatment, whereas triiodothyronine was less potent than tomatidine (Supplementary Fig. 9). Moreover, dexamethasone and fatty acids, but not tomatidine, increased the expression of Connexin 43. Mature CMs exhibit structural changes and increases in size and length compared to hESC-CMs, although they are still smaller than adult CMs^45^. The sarcomeres of immature hESC-CMs have been reported to be 1.6 μm in length, while those of mature CMs are 2~2.3 μm. Engineered cardiac tissues treated with T3, dexamethasone, and insulin-like growth factor 1 exhibit mature sarcomeric Z-line increases in width, alignment, and sarcomere length, although M-lines are invisible^46^. Moreover, engineered cardiac tissues cultured with electromechanical training exhibited mature CM-like phenotypes in terms of physiological sarcomere length (2.2 μm) and M-lines^47^. In the present study, we demonstrated that tomatidine treatment increased the sarcomere length from 1.5 to 1.9 μm in hESC-CMs, supporting the tomatidine-stimulated maturation of hESC-CMs, although the sarcomere length in the Toma-CMs was still shorter than that in previously reported engineered cardiac tissues. These results suggest that the Toma-CMs are still immature compared to adult CMs. For further enhancement of the maturation of hESC-CMs, electromechanical stimuli, cotreatment with other maturation-stimulating agents, such as T3 and dexamethasone, metabolic maturation with fatty acids, and coculture of hESC-CMs with endothelial cells and cardiac fibroblasts may be needed to improve the maturity of hESC-CMs in addition to treatment with tomatidine.

In adult CMs, the resting membrane potential is maintained at approximately 85 mV by the inward-rectifying current I_k1_^48^, which is exerted by the inward-rectifying potassium channels Kir2.1 and Kir2.2 encoded by the genes *KCNJ2* and *KCNJ12*, respectively. I_k1_ stabilizes the resting membrane potential and plays a role in shaping the initial depolarization and final repolarization of the action potential^49^. We found that tomatidine treatment increased the inward-rectifying current I_k1_ and the mRNA levels of *KCNJ2* and *KCNJ12* in hESC-CMs. Furthermore, tomatidine treatment increased the APD in hESC-CMs. The conduction velocity of mature CMs was reported to be 60 cm/s, whereas that of immature CMs was found to be 10–20 cm/s^50^. Tomatidine treatment increased the conduction velocity from ~20 to ~30 cm/s and significantly augmented the spike amplitude and beat period of field potentials. Furthermore, tomatidine treatment enhanced the Ca^2+^ transient in hESC-CMs and reduced the frequency of calcium transients, as evidenced by the increase in Vmax decay and time to decay. These results support tomatidine-induced maturation of the electrophysiological phenotype in hESC-CMs.

The morphology and function of mitochondria are crucial factors for CM maturation. Mitochondria in mature cardiomyocytes exhibit densely organized cristae that house the electron transport chain and ATP synthase^51^. T3 treatment increases maximal oxygen consumption, promotes appropriate muscle responsiveness to insulin, and stimulates oxidative pathways by increasing mitochondrial biogenesis^52^. In the present study, we demonstrated that tomatidine treatment increased the respiratory capacity of hESC-CMs, ATP production, and mitochondrial membrane potential with an increase in the number and size of mitochondria. Moreover, the expression levels of mitochondrial proteins, such as OPA1 and MIC60, and the ratio of mtDNA/nDNA were increased in the Toma-CMs. Whereas mitochondria of the control CMs exhibited small and round morphology, mitochondria of Toma-CMs possessed a long and densely organized cristae structure resembling a cardiac mitochondria phenotype. The metabolic transition from immature CMs to mature CMs is driven by the activation of several transcription factors, including *PPARGC1A/B*, *PPARA*, *NRF1/2*, and *ESRRA/B/G*^53^. Both PPARs and ERRs directly execute the inducible effect of PGC1a/b encoded by *PPARGC1A* and *PPARGC1B* and are master regulators of both oxidative respiration and mitochondrial biogenesis^53^. In addition, T3, dexamethasone, and ursolic acid have been shown to induce mitochondrial biogenesis through the activation of PGC1α^37,54,55^, which plays a key role in high-energy-consuming tissues, including the muscle, heart, and liver^56^. Therefore, it is likely that the tomatidine-stimulated PGC1α pathway promotes mitochondrial biogenesis, which plays a pivotal role in cardiac differentiation.

hESC-CMs are highly useful for cell transplantation in myocardial repair, cardiotoxicity testing during drug development, and patient-specific disease modeling. Dox has been reported to induce cardiotoxicity, leading to heart failure in a subset of patients. Dox causes cell death, mitochondrial dysfunction, and an increase in ROS and intracellular calcium concentrations^57^. A previous study reported that hiPSC-CMs cultured for 60 days produced higher levels of ROS than those cultured for 30 days^13^. The Day 60 hiPSC-CMs had more mature phenotypes in terms of gene expression, myofilament structure, gap junctions, and calcium transients and could more accurately mimic Dox-induced cardiotoxicity in the pathophysiological process of DNA damage. In the present study, we demonstrated that the Toma-CMs were more sensitive to Dox-induced cell death than the control CMs. Dox treatment significantly reduced the beat period and spike amplitude and increased the spike slope in the Toma-CMs compared with the control CMs. These results suggest that the tomatidine-induced maturation of hESC-CMs provides a useful model for the evaluation of cardiotoxicity. To evaluate these benefits, researchers will need to investigate the effects of cardiotoxic drugs in Toma-CMs.

In summary, the present study demonstrates, for the first time, that tomatidine induces the maturation of hESC-CMs to adult CM-like cell types. Toma-CMs exhibit mature CM-like structural phenotypes, electrophysiological characteristics, and enhanced mitochondrial function. Mature CMs provide a valuable platform for cardiotoxicity testing and patient-specific disease modeling, including that of mitochondrial diseases.

## Supplementary information

Supplementary Materials

## Data Availability

All other data are included in the article or Supplementary Information and available from the authors on request.
