# Supplementary material for: Tomatidine-stimulated maturation of human embryonic stem cell-derived cardiomyocytes for modeling mitochondrial dysfunction
Source: Exp Mol Med. 2022 Apr 4;54(4):493–502. doi: 10.1038/s12276-022-00746-8 (PMC9076832; doi:10.1038/s12276-022-00746-8)
Supplement: Supplementary file 1 — Supplementary Materials [file 12276_2022_746_MOESM1_ESM.pdf]

Supporting Information

Tomatidine-stimulated Maturation of Human Embryonic Stem Cell-derived Cardiomyocytes for Modeling Mitochondrial Dysfunction

Ye Seul Kim[1*], Jung Won Yoon[1], Da Sol Kim[1], Seung Hak Choi[2], Hyoung Kyu Kim[2], Jae Boum Youm[2], Jin Han[2], Soon Chul Heo[3], Sung-Ae Hyun[4], Jung-Wook Seo[4], Deok-Ho Kim[5], Jae Ho Kim[1¶]

[1]Department of Physiology, School of Medicine, Pusan National University, Yangsan, Republic of Korea; [2]Department of Physiology and Biophysics, College of Medicine, Cardiovascular and Metabolic Disease Center, Inje University, Busan, Republic of Korea; [3]Department of Oral Physiology, School of Dentistry, Pusan National University, Yangsan, Republic of Korea; [4]Safety Pharmacology Research Group, Korea Institute of Toxicology, Daejeon 34114, Republic of Korea; [5]Department of Biomedical Engineering, Johns Hopkins University, Baltimore, MD 21205, United States

Appendices

Appendix A. Expanded Methods

Appendix B. Supplementary Fig.

Appendix C. Supplementary Table

Expanded Methods

Immunoblotting

For western blotting analysis, cells were lysed in lysis buffer (20 mM Tris-HCl, 1 mM EDTA, 1 mM EGTA, 10 mM NaCl, 1 mM Na_3VO_4, 0.1 mM phenylmethylsulfonyl fluoride, 30 mM sodium pyrophosphate, 25 mM β-glycerol phosphate, 1% Triton X-100, and pH 7.4). Briefly, the cell lysates were loaded on 8–15% tris-glycine SDS-PAGE gels and transferred onto nitrocellulose membranes and/or PVDF membranes. After staining with 0.1% Ponceau S solution (Sigma-Aldrich, #P3504), the membranes were blocked with 5% non-fat milk for 1 h. The membranes were incubated with primary antibodies overnight at 4 °C. After overnight incubation, the membrane was washed four times with 1× TBST buffer to remove excess primary antibody, and appropriate secondary antibodies were diluted in 5% instant skimmed milk for 2 h at room temperature. Finally, the membrane was developed using a chemiluminescence western blotting system (ECL; Amersham Biosciences, Piscataway, NJ, USA).

Quantitative real time PCR (qRT-PCR) analysis

Total RNA was extracted using the TRIsure™ (#BIO-38033; Meridian Bioscience, Cincinnati, Ohio, USA) reagent. cDNA was prepared using a reverse transcription cDNA kit (#RT50KN; NanoHelix, Daejeon, South Korea,). PCR amplifications were carried out in 96-well optical plates with a 20 μL reaction volume, consisting of 20 ng cDNA template, 4 pmol primers, and BrightGreen 2× qPCR MasterMix (#MasterMix-LR; Applied Biological Materials, Richmond, BC, Canada). qRT-PCR was performed using an ABI7500 system (Applied Biosystems, Foster City, CA, USA). Experiments were performed in triplicate and relative mRNA expression was normalized to GAPDH expression. Data were analyzed using the △△CT method and normalized to GAPDH. Primer sequences for reverse transcription-polymerase chain reaction

analysis of gene expression were listed in the Supplementary Table 2.

Flow cytometry analysis

For flow cytometry analysis, the hESC-CMs were dissociated by treatment with Accutase, fixed with 4% paraformaldehyde for 15 min, washed with HBSS, and permeabilized in PBS, containing 1% Triton X-100, for 10 min. The cells were incubated in PBS, supplemented with 5% BSA and 0.1% Tween-20, for 2 h at room temperature(25 °C), followed by treatment with primary antibodies overnight at 4 °C. The cells were incubated with Alexa 488 goat anti-mouse or Alexa 647 donkey anti-rabbit secondary antibodies (Thermo Fisher Scientific, Inc.; 1:1,000 dilution) for 2 h at room temperature, and the fluorescence of each cell was analyzed on an Attune NxT (Thermo Fisher Scientific). Statistical analysis was performed using the FlowJo (ver. 10, Tree Star Inc., Ashland, Oregon, USA) program.

Immunochemistry and confocal microscopy

hESC-CMs were fixed in 4% paraformaldehyde solution for 15 min, permeabilized in 1× TBST buffer, containing 0.1% Tween-20, for 10 min, blocked with 5% BSA (#A7946, Sigma) for 2 h, and incubated with the primary antibodies overnight at 4 °C. The cells were then incubated with blocking buffer, containing the secondary antibodies conjugated with Alexa fluor 488 or 647 nm dyes, at room temperature for 2 h. Cells were mounted with Vectashield medium (#H-1200; Vector Labs, Burlingame, CA, USA), containing 4',6-diamidino-2-phenylindole (DAPI), for DNA staining. The fluorescence images were captured using a confocal microscope system (FluoView FV1000, Olympus Corp., Tokyo, Japan).

Measurement of calcium transients

Intracellular calcium transient was measured in hESC-CMs in the absence and presence of

tomatidine. hESC-CMs were incubated with 2 μM cell permeant ratiometric calcium sensitive dye Fluo-4AM (#F14201, Invitrogen) for 30 min at 37 °C. The cells were washed thrice. Spontaneous calcium release from intracellular stores was measured without electrical stimulation. Calcium transients were continuously recorded using a DeltaVision Ultra (GE Healthcare, Chicago, Illinois, USA) for 1 min. We analyzed the amplitudes of calcium transients as the background corrected pseudoration (△F/F) = (F-F_{base})/(F_{base}-B), where F and F_{base} are the maximum and minimum fluorescence intensities, respectively, and B is the average background signal from areas from the targeted cell. The velocity of transients rate (upstroke of Vmax), time to decay, and transient decay (Decay Vmax) were subsequently calculated and analyzed using the ImageJ software (https://imagej.nih.gov/ij/).

Analysis of mtDNA/nDNA ratio

To prepare total DNA, including mitochondrial DNA (mtDNA), hESC-CMs were washed with HBSS and lysed in 500 μL lysis buffer (10 mM Tris, 1 mM EDTA, 0.3 M NaAc, 1% SDS, and pH 8) with 5 μL proteinase K (Sigma-Aldrich, #T4850) by incubating overnight at 37 °C. Next day, the lysates were treated with 0.2 mg/mL RNase A for 30 min at 37 °C to decompose all fragments except DNA from intracellular extracts. To extract nuclear DNA (nDNA) and mtDNA, 500 μL PCI solution (with phenol, chloroform, and isoamyl alcohol; 25:24:1, #P3803, Sigma-Aldrich) was added to the lysates. The upper-phase solution was transferred to a new tube to obtain precipitated DNA, and absolute EtOH containing 0.3 M sodium acetate (pH 5.2) was added to precipitate the DNA. Finally, for DNA extraction, the precipitated DNA was dissolved in 50 μL nuclease-free water. To determine the ratio of mtDNA/nDNA, qRT-PCR was performed on an ABI 7500 using the BrightGreen 2× qPCR MasterMix with the primers listed in Supplementary Table 3.

Transmission electron microscopy

Cardiomyocytes, with or without tomatidine treatment, were pelleted and fixed in 2% PFA, with 2.5% glutaraldehyde in 0.15 M cacodylate buffer and pH 7.4, overnight at 4 °C. The samples were washed thrice for 10 min each in cacodylate buffer and incubated for 2 h in the dark in 2% osmium tetroxide/cacodylate buffer (OsO_4). Samples were washed thrice for 10 min each in cacodylate buffer and twice for 10 min each in ethanol (two times for 10 min, 50%; two times for 10 min, 60%; two times for 10 min, 70%; two times for 10 min, 80%; two times for 10 min, 90%; two times for 10 min, 95%; three times for 10 min, 100%) and propylene oxide (two times for 10 min), and preincubated in 3:1 propylene oxide/Epon (2 h), 2:1 propylene oxide/Epon (2 h), 1:1 propylene oxide/Epon (2 h), and pure Epon, overnight. Finally, the samples were embedded in Epon 812 resin and hardened at 60 °C for 48 h. Ultrathin sections (100 nm) were then generated and placed on hexagonal copper grids. After sample contrasting, the sections were dried and TEM images were captured using a Tecnai G2 TEM operated by Brain Research Core Facilities at the Korea Brain Research Institute (Daegu, Republic of Korea).

Measurement of mitochondrial membrane potential

Mitochondrial membrane potential (MMP) was measured using the TMRM dye (Invitrogen). Briefly, hESC-CMs were cultured in 12-well plates, washed twice with PBS, and treated with 500 µL of the TMRM (50 nM) working solution for 30 min at 37 °C. The stained cells were washed twice and collected in the dark. After washing twice with PBS, the cells were analyzed with flow cytometry and fluorescence microscopy.

Measurement of mitochondrial superoxide content

Mitochondrial superoxide content was measured using the MitoSOX Red mitochondrial

superoxide indicator (Molecular Probes, Invitrogen). hESC-CMs were seeded on 0.1% gelatin-coated glass coverslips and loaded with 5 μM MitoSOX Red for 20 min at 37 °C, and coverslips were mounted. Images were obtained using an Olympus FV-1000 microscope.

T-tubule formation analysis

T-tubule analysis was performed by incubating live hESC-CMs with 2 μM Di-8-ANEPPS (Thermo Fisher) in cell growth medium for 20 min at 37 °C. The cells were washed twice with dPBS. Di-8-ANEPPS were excited using a 488 nm laser and emission with a 595/50 filter. Images was observed using an Olympus FV1000 microscope.

Measurement of ATP levels

Cellular ATP levels were determined using a CellTiter-Glo (#G7571, Promega) luminescent cell viability assay kit, which can be used to determine the amount of ATP in cells. hESC-CMs were cultured in multi-well plates, and ATP levels were determined using the CellTiter-Glo assay kit according to the manufacturer’s protocol. ATP content of the samples was calculated using the value of recorded luminescence.

Electrophysiological analysis

Whole-cell patch-clamp recordings were performed with hESC-CMs placed in a chamber mounted on the stage of an inverted microscope by using the Axopatch 200 B amplifier (Axon Instrument, Molecular Devices, San Jose, CA, USA) at room temperature (22 ± 1 °C). Patch pipettes were pulled from thin-walled borosilicate capillaries (Clark Electromedical Instruments, Pangbourne, UK) using a PP-83 vertical puller (Narishige Scientific Instrument Laboratory, Tokyo, Japan). During control experiments, hESC-CMs were continuously perfused with bath solution: 130 mM NaCl, 5.9 mM KCl, 1.2 mM $MgCl_2$, 10 mM HEPES, 11

mM glucose, and 2.4 mM $CaCl_2$ (pH 7.4 with NaOH). The pipette was filled with pipette solution: 6 mM NaCl, 126 mM KCl, 1 mM MgATP, 1.2 mM $MgCl_2$, 10 mM HEPES, 5 mM ethylene glycol-bis (β-aminoethyl ether)-N,N,N',N'-tetra acetic acid (EGTA), and 11 mM glucose (pH 7.4 with KOH). For isolation of the inward-rectifying K^+ currents, 0.5 mM $BaCl_2$ was used. Whole-cell patches with a seal resistance, between patch pipette and cell membrane, higher than 2 GΩ were chosen to record whole-cell membrane currents. The access resistances of the patch pipettes were all less than 5 MΩ with a normal K^+-rich pipette filling solution. The liquid-junction potentials between bathing and pipette filling solution based on ionic mobility were lower than 5 mV. All recordings were carried out at least 5 min after achieving whole-cell configuration for stabilization. Membrane currents were filtered at 10 kHz, with a 4-pole Bessel-type low-pass filter, and sampled at a rate of 27 kHz. All experimental parameters, such as pulse generation and data acquisition, were controlled using our home-made software (PatchPro®).

Statistical analysis

All grouped data are presented as mean ± S.D. ($n \geq 3$). Statistical significance was determined using one-way ANOVA, two-way ANOVA, and two-tailed unpaired Student's *t*-tests.

Supplementary Figures

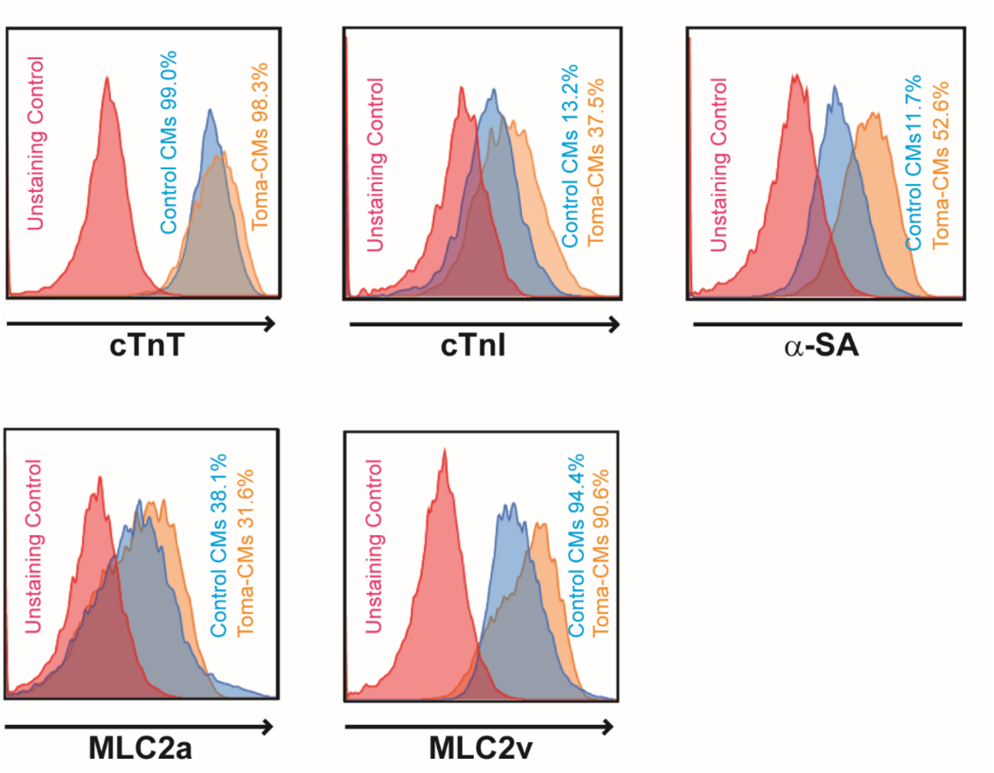

Supplementary Fig. 1. FACS analysis of cardiomyocyte markers. Representative FACS analysis data of cardiomyocyte markers in control CMs and Toma-CMs. After cardiomyocyte selection with lactic acid treatment, day 30 control CM and Toma-CMs were incubated with different antibodies against cTnT, cTnI, α-SA, MLC2a and MLC2v. Staining with the appropriate IgG isotype control antibody is shown in red. The percentages of each marker in control CM and Toma-CM are shown.

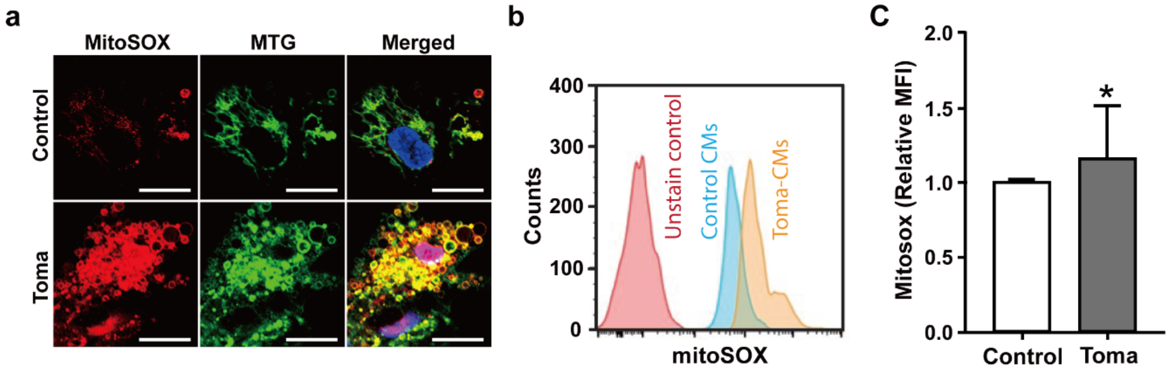

Supplementary Fig. 2. Increased mitochondrial superoxide generation in Toma-CMs. (a) Representative confocal images of mitochondrial superoxide in hESC-CMs. Control CMs and Toma-CMs were incubated with MitoSOX^{TM} and MitoTracker^{TM} green (MTG), and nuclei were stained blue with DAPI. MitoSOX (red color) and MitoTracker (green color) specifically stained mitochondrial superoxide and mitochondria. (b) Flow cytometry plots of MitoSOX in control CM and Toma-CM. Staining with the appropriate IgG isotype control antibody is shown in red. (c) The mean values of MitoSOX intensity are shown for control CM and Toma-CM (MFI; mean fluorescent intensity, n = 4). Scale bar = 100 μm. *, p<0.05

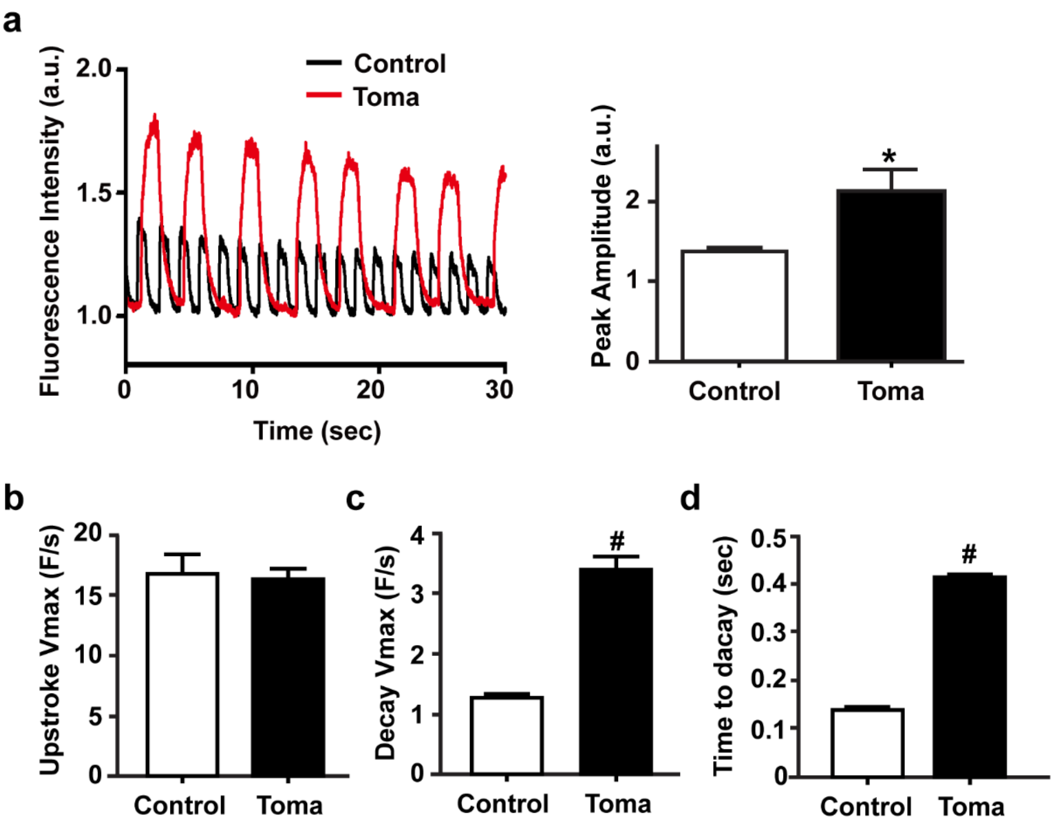

Supplementary Fig. 3. Enhanced calcium transients in Toma-CMs. (a) Representative trace of calcium transients in control CMs and Toma-CMs. Calcium transients were measured with cell permeable calcium sensitive dye Fluo-4AM. Peak amplitude was measured from the fluorescence intensity of calcium transient. Quantification of (b) maximal upstroke velocity (V_{max} upstroke), (c) maximal upstroke velocity (V_{max} decay), and (d) time to decay of calcium transients in hESC-CMs. Unpaired *t*-test was performed between control and Toma-CM (n = 17), *, $p < 0.05$; #, $p < 0.001$.

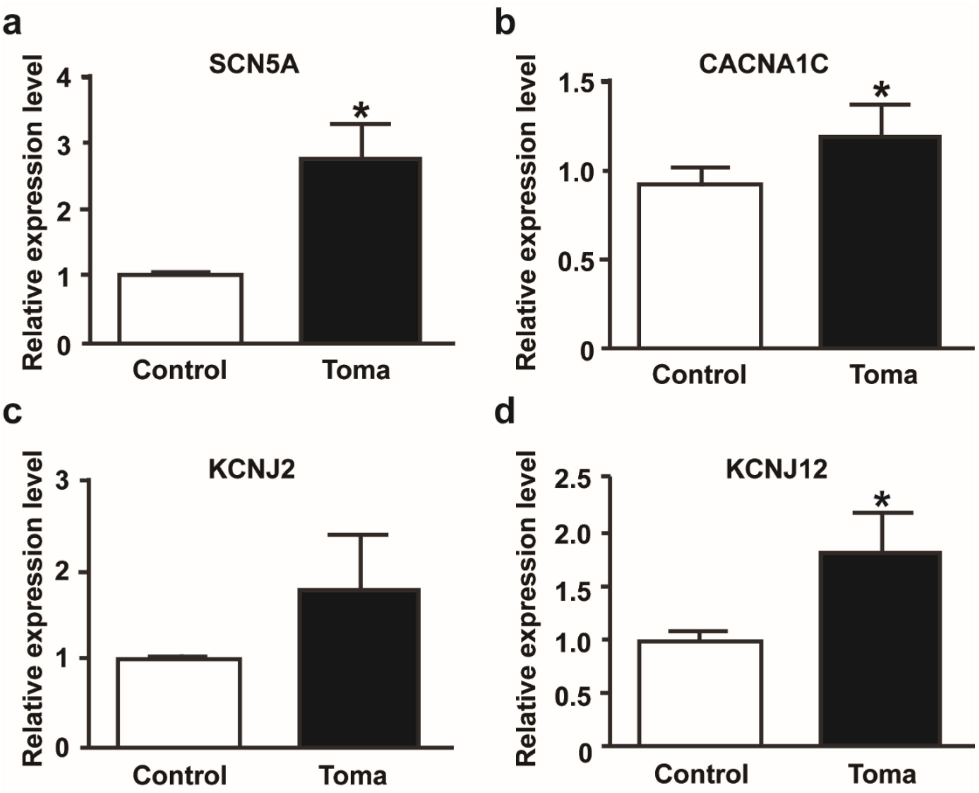

Supplementary Fig. 4. Increased expression of cardiomyocyte-specific ion channels in control and Toma-CM. The expression levels of (a) SCN5A (Nav 1.5), (b) CACNA1C (Cav 1.2), (c) KCNJ2 (Kir2.1), and (d) KCNJ12 (Kir2.2) subtypes in control CM and Toma-CM were accessed by qPCR. qPCR data are represented as mean ± S.D. (n = 9 - 16) . *, p<0.05 vs control CM.

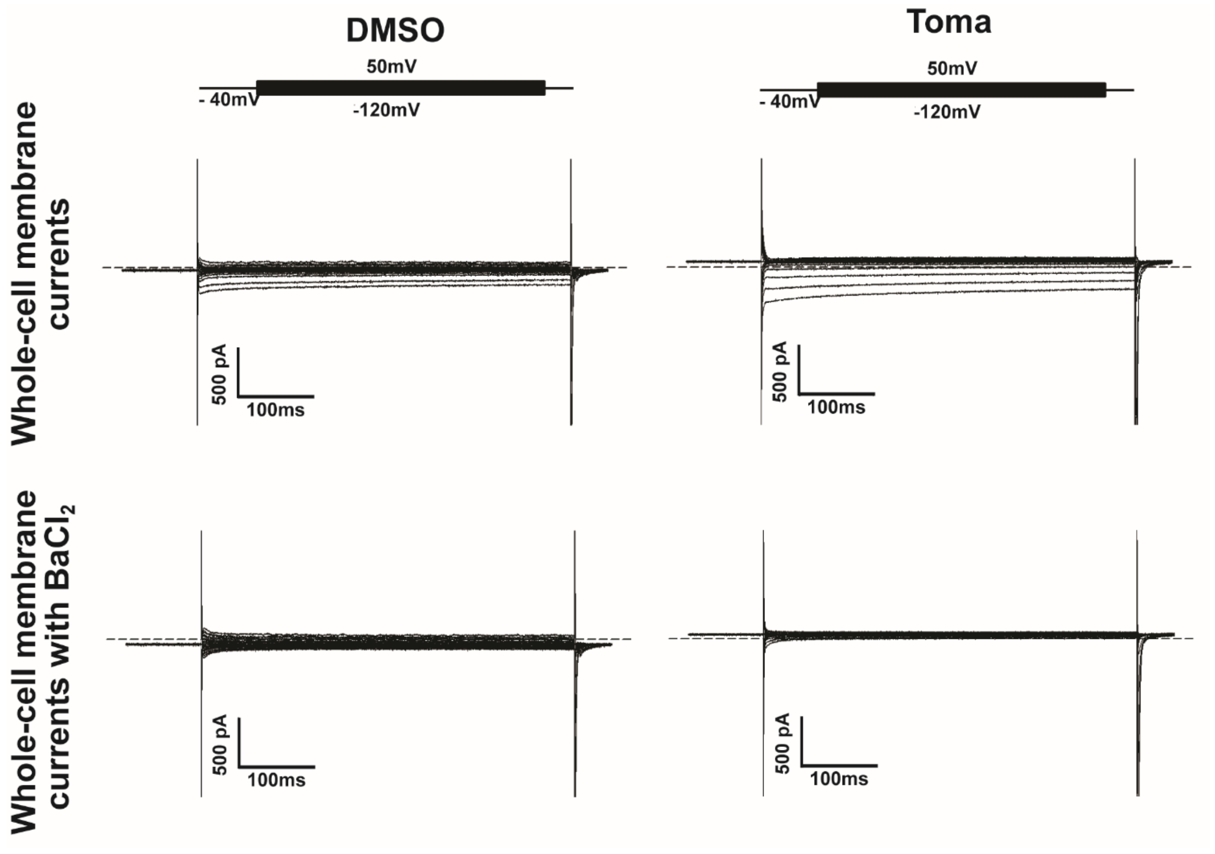

Supplementary Fig. 5. Inward-rectifying K^+ currents in control CMs and Toma-CMs. Top and Bottom panels indicate whole-cell membrane currents in the absence and presence of $BaCl_2$, respectively. Representative membrane currents were all obtained by averaging the results from 7 different cells.

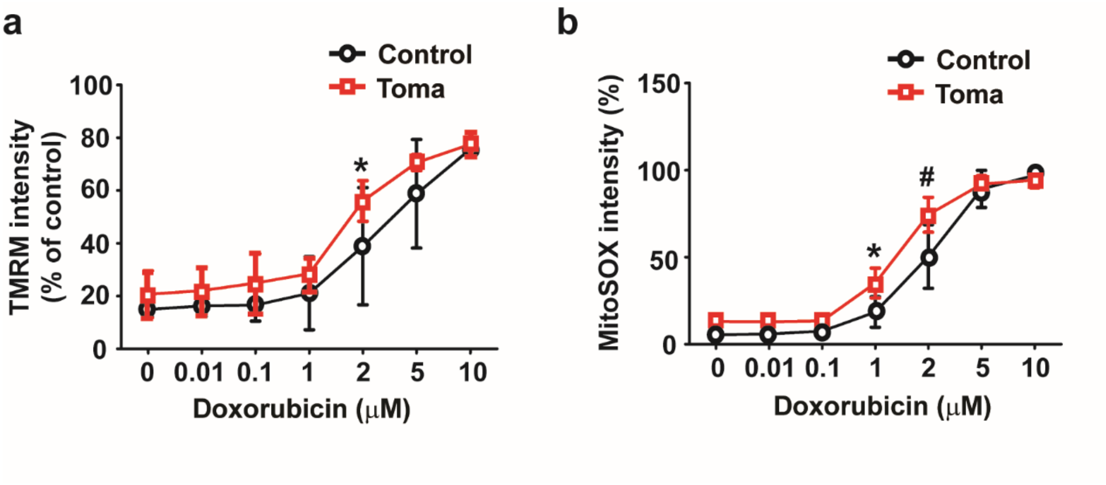

Supplementary Fig. 6. Effects of Doxorubicin on mitochondrial membrane potentials and superoxide in Toma-CMs. Control CMs and Toma-CMs were treated with increasing concentrations of doxorubicin, mitochondrial membrane potential intensity (a) and mitochondrial superoxide (b) were measured by flow cytometry analysis. (n = 8). Two-way ANOVA, *, $p < 0.05$; #, $p < 0.001$ *vs* control CM.

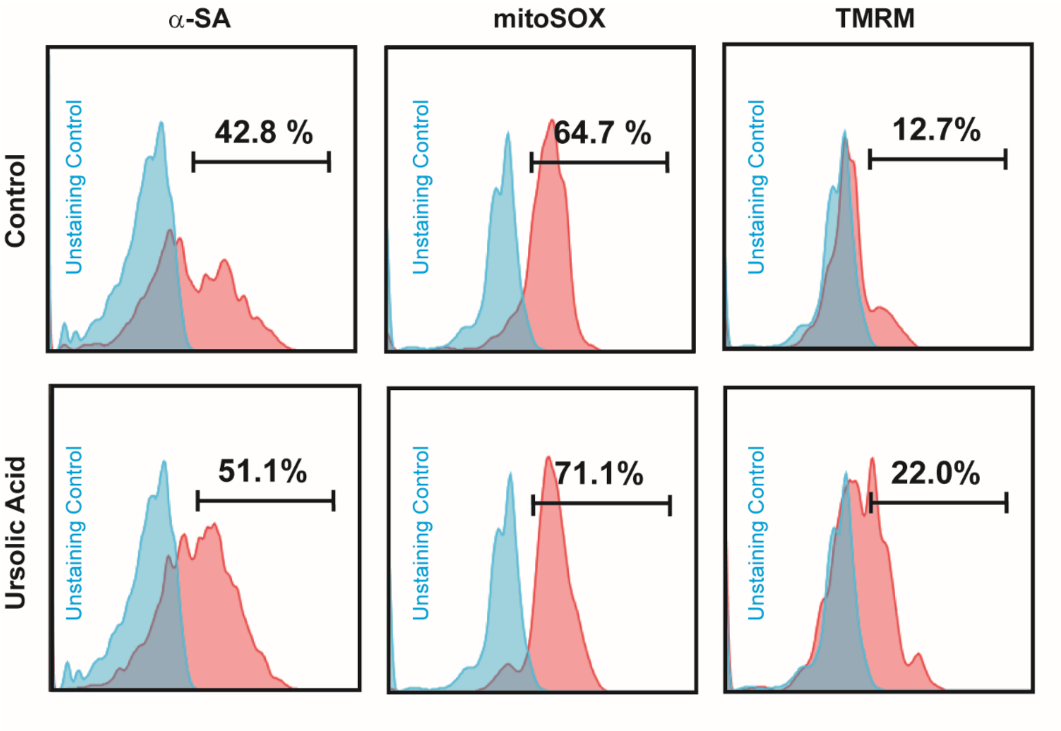

Supplementary Fig. 7. Effects of Ursolic acid on α-SA expression, mitochondrial superoxide, and mitochondrial membrane potential in hESC-CMs. hESC-CMs were treated with DMSO or 1 μM ursolic acid, and the day 30 hESC-CMs were subjected to FACS analysis. Representative FACS plots of α-SA, MitoSOX, and TMRM in DMSO- and Ursolic acid- treated hESC-CMs are shown. Cells were stained with the appropriate IgG isotype control antibody, and the percentages of cells positive for α-SA, MitoSOX, and TMRM are shown.

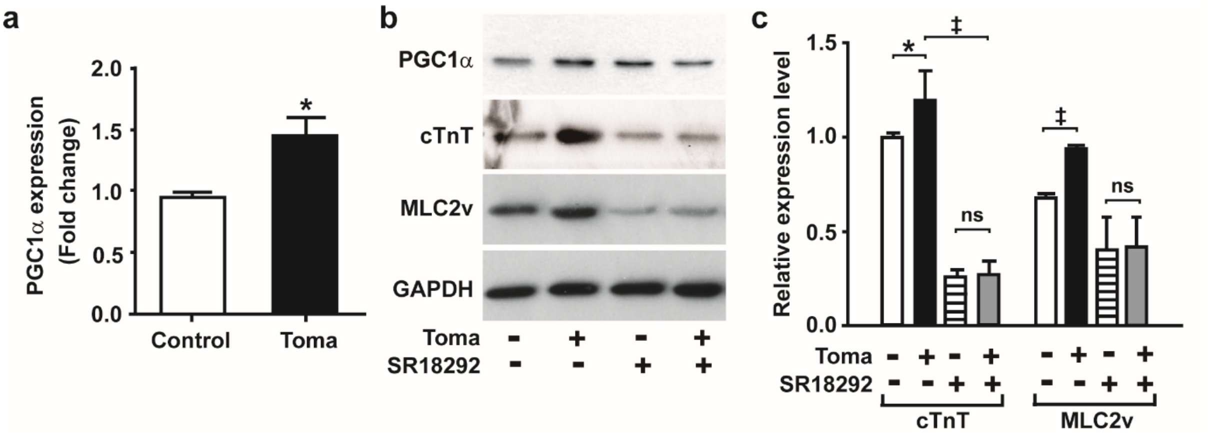

Supplementary Fig. 8. Role of PGC1α on tomatidine-stimulated cardiac differentiation of hESC-CMs. (a) The expression of PGC1α in control CM and Toma-CM was quantified by western blotting. (b) hESC-CMs were treated with 1 μM tomatidine or control (DMSO) in the absence or presence of 20 μM SR18292, and the expression of PGC1α, cTnT, MLC2v, and GAPDH was determined by western blotting. (c) The protein levels of cTnT and MLC2v in 60 day hESC-CMs were quantified from the panel B. Data are shown as the mean ± S.D. (n = 4), unpaired student's t-test, *, $p < 0.05$; ‡, $p < 0.01$.

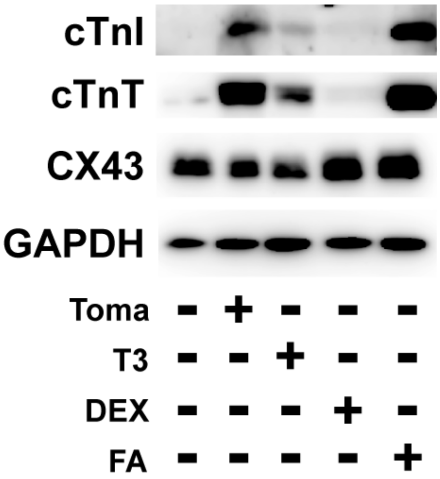

Supplementary Fig. 9. Effects of tomatidine, T3, dexamethasone, and fatty acids on cardiomyocyte differentiation of hESCs. hESC-CMs were treated with 1 µM tomatidine, 0.1 µM triiodothyronine (T3), 1 µM dexamethasone (DEX), and fatty acid (FA)-albumin complex (105 µM palmitate, 81 µM oleic acid, and 45 µM linoleic acid) for a week. The protein levels of cTnI, cTnT, Connexin 43 (Cx43), and GAPDH were determined by western blot analysis.

Supplementary Tables

Supplementary Table 1. List of primary antibodies used in this study

Antibody	Supplier	Catalog number	Host	Dilution
GAPDH	Santa Cruz Biotechnology	SC-47724	Mouse	1:2000
α-SA	Sigma-Aldrich	A7732	Mouse	1:2000
cTnT	Abcam	Ab45932	Rabbit	1:5000
MLC2a	Synaptic Systems GmbH	311 011	Mouse	1:500
MLC2v	Thermofisher Scientific	10906-1-ap	Rabbit	1:500
cTnI	Abcam	Ab47003	Rabbit	1:2000
BIN1	Abcam	Ab185950	Rabbit	1:500
JPH2	Biorbyt	Orb13188	Mouse	1:250
AMPK	Cell Signaling Technology	#5832	Rabbit	1:1000
p-AMPK (Thr172)	Cell Signaling Technology	#2535	Rabbit	1:1000
PGC1a	Novus Biologicals	NBP1-04676	Rabbit	1:500
OPA1	Abcam	Ab42364	Rabbit	1:1000
Tom20	Santa Cruz Biotechnology	Sc-17764	Mouse	1:500
Mic60	Abcam	Ab110329	Mouse	1:1000

Supplementary Table 2. Primer sequences of RT-PCR analysis

Gene symbol	Forward Primers (5’-3’)	Reverse Primers (5’-3’)
TNNI3	CACCTCAAGCAGGTGAAGAAG	CAGGAAGGCTCAGCTCTCAA
MYL2	TACGTTCGGGAAATGCTGAC	TTCTCCGTGGGTGATGATG
MYL7	CCGTCTTCCTCACGCTCTT	TGAACTCATCCTTGTTCACCAC
MYH7	AGTCCCAGGTCAACAAGCTG	GGGCTGAGCAGATCAAGATG
MYH6	CCAGGTCAACAAGCTTCGAG	TGTCACTCCTCATCGTGCAT
SCN5A	GAGCTCTGTCACGATTTGAGG	GAAGATGAGGCAGACGAGGA
CACNA1C	CAATCTCCGAAGAGGGGTTT	TCGCTTCAGACATTCCAGGT
KCNJ12	TGGATCCTTTCCAGTTGGTG	CGGCTCCTCTTGAGTTCTATCTT
KCNJ2	CGCTTTTTACAAACCACTGGA	CGGCTCCTCTTGAGTTCTATCTT

Supplementary Table 3. Primer sequences for RT-PCR analysis of mtDNA and ncDNA

Gene symbol	Forward Primers (5’-3’)	Reverse Primers (5’-3’)
COX2	AGTACACCGACTACGGCGGAC	TCGGGAGTACTACTCGATTGTC
16sRibosome	ACCTAACAAACCCACAGGTCC	CTTAGCATGTACTGCCGGAG
HK2	TTCACTTCTTGGTCCCTTTCCA	ACTCACCCCTCTCCTCTGGAT
UCP2	GTGTGATGGGCACCATTCTGAC	TGTTTGACAGAATCATACAGGCC
